# Cascade Screening for Fragile X Syndrome/CGG Repeat Expansions in Children Attending Special Education in Sri Lanka

**DOI:** 10.1371/journal.pone.0145537

**Published:** 2015-12-22

**Authors:** C. H. W. M. R. Bhagya Chandrasekara, W. S. Sulochana Wijesundera, Hemamali N. Perera, Samuel S. Chong, Indhu-Shree Rajan-Babu

**Affiliations:** 1 Department of Biochemistry and Molecular Biology, Faculty of Medicine, University of Colombo, Kynsey road, Colombo 08, Sri Lanka; 2 Department of Psychological Medicine, Faculty of Medicine, University of Colombo, Kynsey road, Colombo 08, Sri Lanka; 3 Department of Pediatrics, Yong Loo Lin School of Medicine, National University of Singapore, Singapore, 119074, Singapore; 4 Khoo Teck Puat - National University Children’s Medical Institute, National University Health System, Singapore, 119228, Singapore; 5 Department of Laboratory Medicine, National University Hospital, Singapore, 119074, Singapore; Institute of Molecular Genetics IMG-CNR, ITALY

## Abstract

Fragile X syndrome (FXS) is the commonest cause of inherited mental retardation and clinically presents with learning, emotional and behaviour problems. FXS is caused by expansion of cytosine-guanine-guanine (CGG) repeats present in the 5’ untranslated region of the *FMR1* gene. The aim of this study was to screen children attending special education institutions in Sri Lanka to estimate the prevalence of CGG repeat expansions. The study population comprised a representative national sample of 850 children (540 males, 310 females) with 5 to 18 years of age from moderate to severe mental retardation of wide ranging aetiology. Screening for CGG repeat expansion was carried out on DNA extracted from buccal cells using 3’ direct triplet primed PCR followed by melting curve analysis. To identify the expanded status of screened positive samples, capillary electrophoresis, methylation specific PCR and Southern hybridization were carried out using venous blood samples. Prevalence of CGG repeat expansions was 2.2%. Further classification of the positive samples into FXS full mutation, pre-mutation and grey zone gave prevalence of 1.3%, 0.8% and 0.1% respectively. All positive cases were male. No females with FXS were detected in our study may have been due to the small sample size.

## Introduction

Fragile X syndrome (FXS) is the commonest cause of inherited mental retardation [1]. It is associated with mild to severe mental retardation (MR), learning disability (LD), behavioural and emotional problems [2]. Molecular basis of FXS is the expansion of cytosine-guanine-guanine (CGG) repeats present in 5’ untranslated region of fragile X mental retardation 1 (*FMR1*) gene. Based on the number of CGG repeats, they are classified as normal (NL, 5–44), grey zone (GZ, 45–54), pre-mutation (PM, 55–200) and full mutation (FM, >200) [3]. CGG repeats greater than 200 are associated with methylation of C_P_G (cytosine, guanine) islands in the *FMR1* gene [4, 5]. Methylation of C_P_G island in the promoter region causes transcriptional silencing of the gene resulting FXS [4]. In addition, a FM with unmethylated C_P_G island in the promoter and PM with methylated C_P_G island in the exon1/intron1 boundary of the *FMR1* gene have also been reported in literature [6, 7].

Screening for the presence of CGG expanded repeats in children is important for providing appropriate education plans. Further, individuals with PM are carriers. Although alleles containing CGG repeats less than 54 are considered as stable, there is evidence where some GZ alleles expand to FM within two generations [8–10]. Majority of the carriers are unaware of their genetic status and associated reproductive risks. Approximately 8% of females and 40% of males with PM are at risk of developing fragile X associated tremor/ataxia syndrome (FXTAS). In addition, 20% of females with PM develop premature ovarian failure (POF) [11–14]. The risk of these PM related disorders increase with age and in relation to the number of CGG repeats in the *FMR1* gene [15]. Accordingly, screening will create an opportunity for prevention. Also, the morphological features described in FXS are unreliable and requires genetic screening to make a definitive diagnosis [16–18]. The aim of this study was to screen children attending special education schools in Sri Lanka to assess the prevalence of CGG repeat expansions. In Sri Lanka, children who are unable to function in mainstream education attend special education institutions. These institutions have countrywide infrastructure and accommodate children up to 18 years of age with moderate to severe mental retardation of wide ranging etiology. The prevalence of FXS has not been studied in Sri Lanka.

Screening and diagnostic methods used worldwide, individually or in combination for detecting CGG repeat expansions include cytogenetic analysis, polymerase chain reaction (PCR), methylation specific PCR (MS-PCR), 3’ and 5’ direct triplet-primed PCR (dTP-PCR) followed by melting curve analysis (MCA), capillary electrophoresis (CE) and Southern hybridization [19–25]. Each method has its inherent advantages and disadvantages. The present study describes the screening of CGG repeat expansions in the *FMR1* gene by amplifying the repeat region using 3’ dTP-PCR followed by MCA on Real Time PCR system [22]. Further analysis was carried out using CE, MS-PCR and Southern hybridization to identify the expanded status of the gene.

## Materials and Methods

### Study design and selection of study sample

This was a population based cross-sectional study of children attending special education institutions in Sri Lanka. A representative national sample was obtained by multi-level stratified sampling and random selection, using simple random numbers at each level.

The following procedure was used in selecting the study sample. (i) The sample size for the study was calculated on the assumption that the possible prevalence of FXS among children attending special education was 10%. The formula used for calculation was n = Z_1-α_^2^P(1-P)/d^2^ where n is the sample size, Z_1-α_^2^ is the standard variate at p<0.05 at 95% confidence interval, and is equal to 1.96. P is the estimate prevalence of FXS among the study population taken as 10%, d is absolute error or precision taken as 0.05. The value obtained for n (sample size) was 774. However, a total of 850 subjects available at the end of sampling were all incorporated. (ii) The total population of children registered for special education with the Ministry of Education, Sri Lanka, at the time of sampling, was 5960, who are distributed in 25 administrative districts. According to the poverty index (Central Bank, Sri Lanka 2012), the 25 districts were categorized into three clusters, of which, 10 districts were selected using simple random numbers. This yielded a total population of 4605. (iii) Using simple random numbers, 95 schools were identified from the 10 districts for inclusion in the study with a total population of 1525. Following exclusion of children with other disabilities incompatible with FXS, 850 were selected for genetic assay. Exclusion criteria were Down syndrome, cerebral palsy, isolated hearing and visual impairment, and brain insult directly resulting from infection and injury.

A convenient sample of 240 children, 153 males and 87 females, 4 to13 years of age, were selected from children attending normal stream education. This control group was used solely for purpose of eliminating potential false positive results in genetic analysis. An exactly similar method was used at all levels of screening (DNA extraction and genetic screening) in both study and control groups.

### DNA extraction

Buccal swabs for DNA extraction were collected after thoroughly rinsing the mouth to ensure non-contamination with food particles. DNA was extracted from buccal cells as described in Handel *et al*., (2006) with minor modifications [26]. The buccal cells were suspended in a solution of 0.1 x Gitschiez buffer and 0.5% Triton X 100. This was followed by treatment with proteinase K (40 μg/ml), addition of saturated NaCl and centrifugation. Supernatant containing DNA was recovered by ethanol precipitation.

### Method of Assay

Initial genetic screening (n = 850) for FXS was performed using 3’dTP-PCR followed by MCA with DNA extracted from buccal swabs. Individuals positive for expanded repeats (elicited by 3’dTP-PCR and MCA) were analyzed using CE, MS-PCR and Southern hybridization with DNA extracted from venous blood samples as described by Miller *et al*., (1988) [27]. Each assay was verified by commercial preparations of FXS FM and PM DNA samples (NA07537, NA06852, NA06897 NA06896, NA06891, Coriell Institute for Medical Research, USA).

### 3’ Direct triplet-primed PCR and melting curve analysis

3’dTP-PCR was performed by following the user-guide of FastFraX^™^ *FMR1* Identification kit, Biofactory Pte. Ltd,Singapore. The MCA conditions were set up as described in Teo *et al*., (2012) [22]. FastFraX^™^ *FMR1* Identification kit has already been validated for the MCA conditions and data is available in Teo *et al*., (2012) and Lim *et al*., (2015) [22, 28].

A sample DNA with 43 CGG repeats in the *FMR1* gene (Coriell Institute for Medical Research, USA) was selected to establish the threshold temperature to distinguish NL alleles from expanded alleles in MCA. The 43 CGG repeat DNA was used in each run of 3’ dTP-PCR followed MCA and placed in the same well position of the 96 well plates. Melting curve profiles were generated by plotting -dF/dT (negative first derivative of fluorescence over temperature) against T (temperature). Baseline temperature was selected to discriminate NL repeats from GZ, PM and FM.

3’dTP-PCR followed by MCA assay was flagged as having expanded repeats when the melt curve profile of a sample dropped to baseline at a temperature higher than that of the sample DNA with 43 CGG repeats. The control group was also screened by 3’dTP-PCR followed MCA using the 43 CGG repeat DNA as the control.

#### 3’ Direct triplet primed PCR and capillary electrophoresis

3’ dTP-PCR was performed according to manufacturer instructions (FastFraX^™^ *FMR1* sizing kit, BiofactoryPte. Ltd, Singapore). Thermocycling conditions were applied as described by Teo *et al* (2012) [22]. PCR products were resolved in a 3130xl Genetic Analyzer (Applied Biosystems). Electropherograms were analyzed with GeneMapper software (version 4.0; Applied Biosystems).

#### Bisulfite conversion and methylation specific PCR

Bisulfite conversion was performed according to the manufactures instructions (EZ DNA Methyaltion Gold^™^ Kit- Invitrogen USA).

MS-PCR was performed as described by Zhou *et al*., (2004) with three primer pairs, non methylated primers (Non Met PCR), methylated primers (Met-PCR) and methylated triplet primers (mTP-PCR) [20]. All amplified products were separated on ethidium bromide stained agarose gel (1.5%) electrophoresis (70 V for 30 min) and visualized under ultraviolet light.

#### Southern hybridization

Genomic DNA (3–4 μg) was digested with *Eco*RI followed by *Nru*I (Roche Diagnostics, Germany) restriction enzymes. The digested product was separated on 0.8% agarose gel at a voltage of 2v/cm for 16 hours. Thereafter the DNA was capillary transferred on to a nylon membrane and hybridized with StB12.3 [29] digoxigenin labeled probe followed by chemiluminescence detection as described elsewhere [30].

Written informed consent was obtained from parents or guardians at all relevant situations. Ethical approval was granted by Ethics Review Committee, Faculty of Medicine, University of Colombo.

## Results

The representative study-sample of 850 was recruited from national population of children attending special education. Age distribution was 5 to 18 years (mean = 10.4, SD = 3.6). Majority, 540 (63.5%) were male.

**Fig 1:**
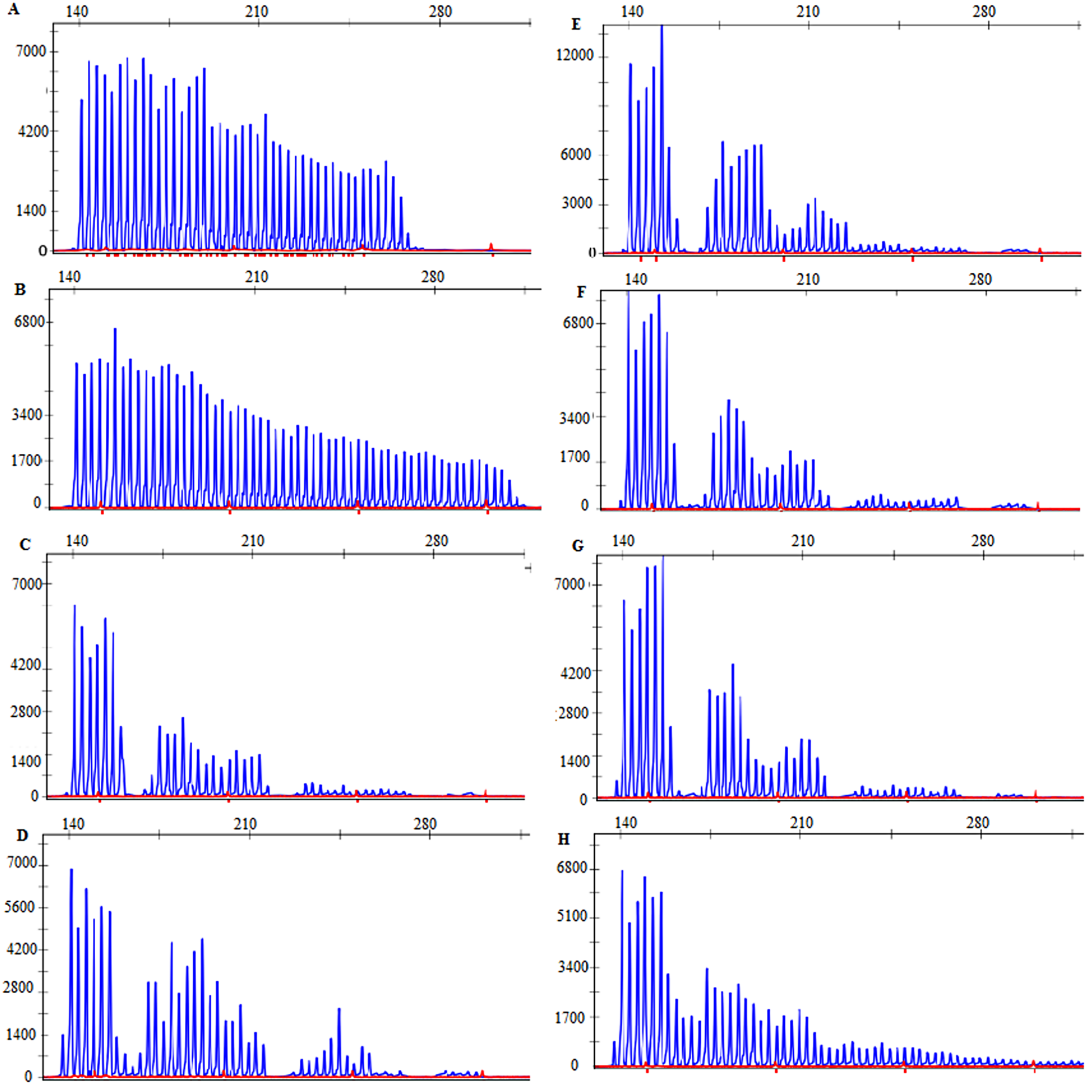
Electropherograms of samples 1–8 obtained from 3’ direct triplet primed PCR followed by capillary electrophoresis. (A)- (H): samples 1 to 8. Peaks in the eletropherogram indicate the number of CGG repeats in each individual.

The 3’dTP-PCR followed by MCA analysis identified 19 (2.2%±0.148), all male sub-sample, having expanded CGG repeats in the *FMR1* gene. Age range was 5 to 16 years (mean = 9.1, SD = 3.5). Results of CE further classified this group into GZ 1(0.1%±0.384), PM 7(0.82%±0.384) and FM 11(1.3%±0.384). Electropherograms of GZ and PM samples are shown in Fig 1. The GZ individual had 47 repeats. Five children with PM had 58 CGG repeats, with the other two having 62 and 140 CGG repeats (Table 1). The profile of CGG repeats of FM individuals could not be clarified using CE alone. Hence, cascade screening using MS-PCR and Southern hybridization was performed. CGG repeat sizes of FM individuals are described later in results.

**Table 1:** CGG repeat sizes and methylation status elicited from capillary electrophoresis, methylation specific PCR and Southern hybridization analysis for the nineteen sub-samples.

Case	Sex/age (years)	Method of analysis			Interpretation
		Capillary Electrophoresis (classification, CGG repeats)	Methylation specific PCR (methylation status, CGG repeats*)	Southern hybridization (fragment size in kb)	
1(#324)	M/8.5	GZ,47	non-methylated,50	~2.9	GZ
2(#414)	M/6	PM,62	non-methylated,60	~2.9	PM
3(#329)	M/3.6	PM,58	non-methylated,60	~2.9	PM
4(#332)	M/10	PM,58	non-methylated,60	~2.9	PM
5(#334)	M/5	PM,58	non-methylated,60	~2.9	PM
6(#885)	M/5	PM,58	non-methylated,60	~2.9	PM
7(#899)	M/5	PM,58	non-methylated,60	~2.9	PM
8(#900)	M/5	PM,140	non-methylated,130	~5.5	PM
9(#95)	M/16	FM	methylated,300	~5.8	FM
10(#217)	M/10	FM	methylated	~6.5	FM
11(#305)	M/12	FM	methylated	~7	FM
12(#318)	M/8.2	Not done	methylated	~7	FM
13(#479)	M/15	FM	methylated	~8	FM
14(#639)	M/11	FM	methylated,mosaic	~6	FM
15(#315)	M/5.8	FM	methylated	~7	FM
16(#333)	M/13	FM	methylated	~7.2	FM
17(#336)	M/12	FM	methylated	~6.3	FM
18(#338)	M/5	FM	methylated,300	~5.8	FM
19(#339)	M/5	FM	methylated	~8	FM

M- male, GZ: grey zone, PM: pre mutation, FM: full mutation,

*: In methylation specific PCR CGG repeats are rounded off to the nearest 10 CGG repeats.

**Fig 2:**
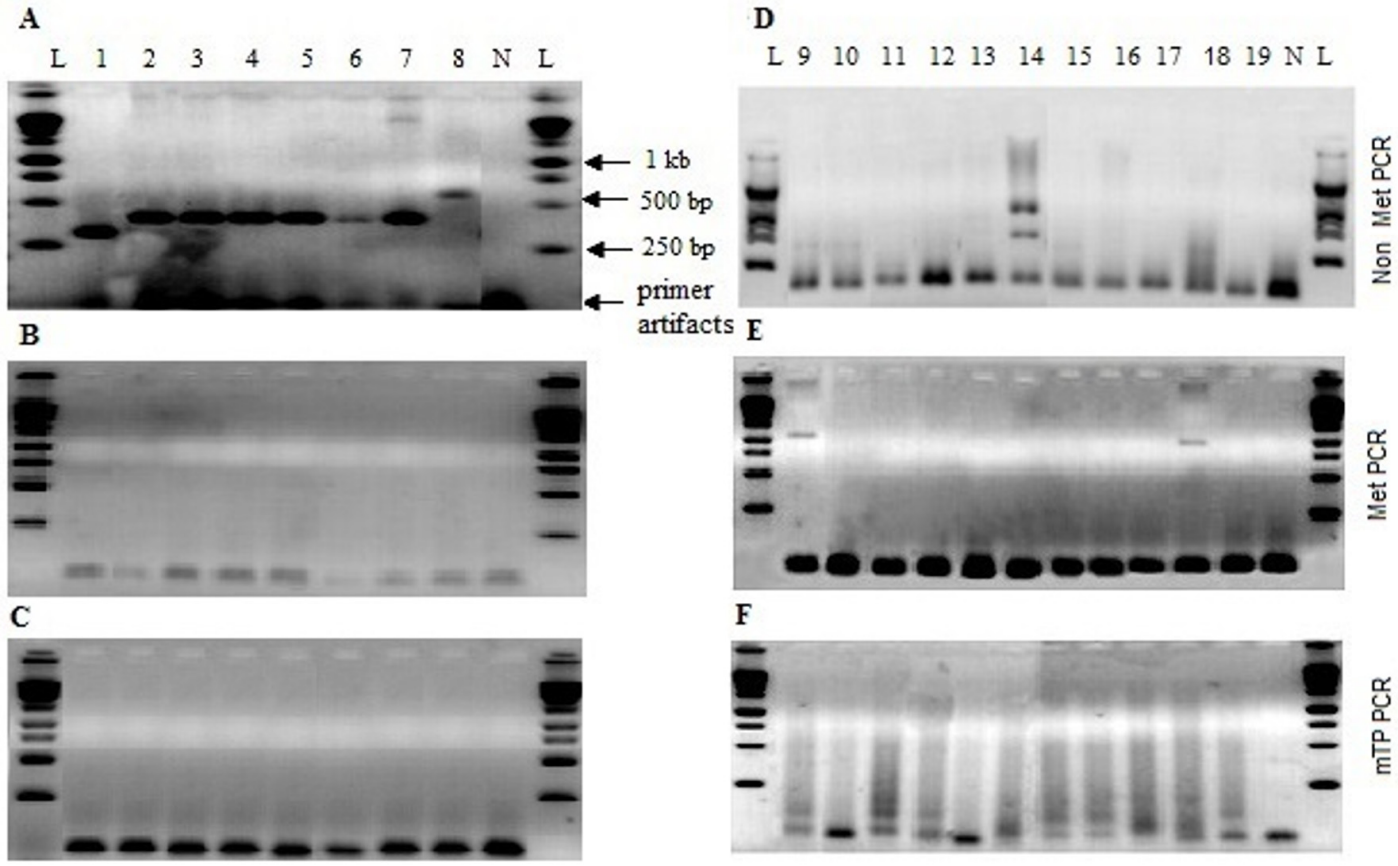
Agarose gel electrophoresis of methylation specific PCR. (A), (B) and (C): samples 1 to 8. (D), (E) and (F): samples 9 to19. Top panel, non methylated PCR (Non Met PCR). Middle panel, methylated PCR (Met PCR). Bottom panel, methylated triplet primed PCR (mTP-PCR). L: l kb DNA molecular weight marker (Promega), N: Negative control (without genomic DNA).

MS-PCR analysis of the 19 samples, having expanded CGG repeats in the *FMR1* gene are shown in Fig 2. The specific findings are as follows.

(i) For samples 1 to 8, Non Met PCR elicited a single PCR fragment around 300 to 550 bp range (Fig 2A) while Met-PCR (Fig 2B) and mTP-PCR (Fig 2C) reactions were negative. (ii) Of the remaining samples (9–19), only sample 14 was positive for Non Met PCR (Fig 2D). (iii) Met PCR resulted ~1kb fragments for samples 9 and 18 revealing the presence of ~300 CGG repeat expansions (Fig 2E). (iv) Absence of Met PCR fragments for samples 10 to 17 and 19 indicated, the presence of CGG repeats greater than 350. (v) Presence of smears in mTP-PCR further confirmed that samples 9 to 19 were FM (Fig 2F). (vi) Sample 14 indicated the presence of mosaicm as it was positive for both Non Met and mTP PCR (Fig 2D and 2F). (vii) The CGG repeat numbers of samples 1 to 9 and 18 were calculated based on the fragment sizes observed in Non Met and Met PCRs using two formulae described in Zhou *et al*., (2004) [20] (Table 1).

**Fig 3:**
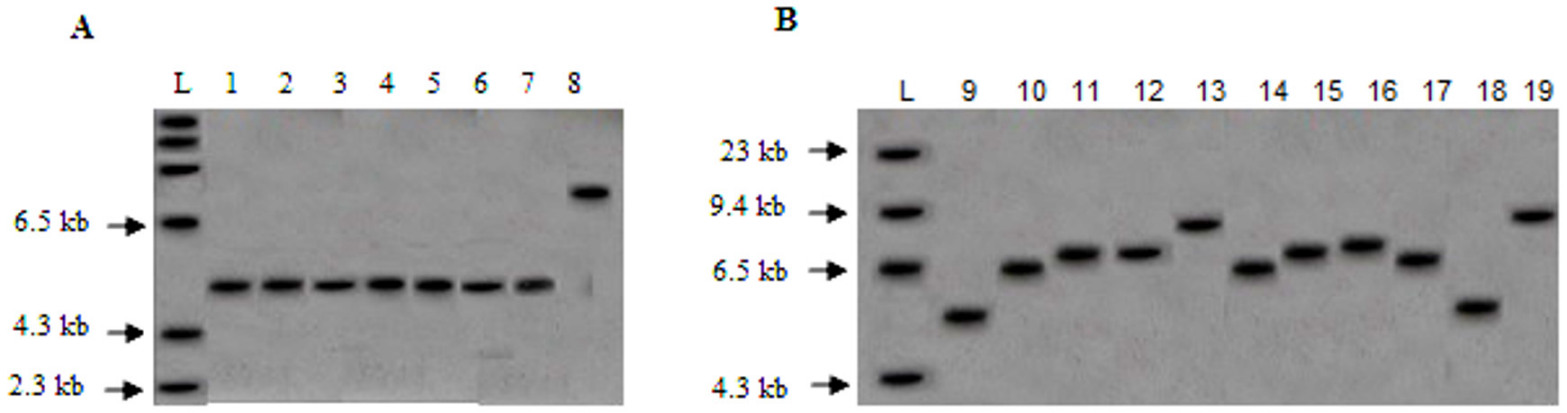
Autoradiogram of Southern hybridization carried on *Eco*RI and *Nru*I digested genomic DNA hybridized with StB12.3 digoxigenin labeled probe. (A): samples 1 to 8. (B): samples 9 to19. L-DNA molecular weight marker II dig labeled (Roche).

In Southern hybridization, repeat sizes and methylation status of the C_P_G island located in the promoter were based on fragment sizes obtained from *Eco*RI/*Nru*I digests hybridized with the probe StB12.3. Southern hybridization showed fragments around 2.9 kb for samples 1 to 7 and fragments in the range of 5.5 kb to 8 kb for samples 8 to 19 (Table 1). These results indicated that samples 1 to 7 have non-methylated C_P_G island while in samples 8–19 the C_P_G island of *FMR1* gene promoter was methylated. However, MS-PCR analysis of sample 8 revealed that the 5’ untranslated repeat region was not methylated. The largest FM identified was ~8 kb having ~ 950 CGG repeats (sample 13 and 19) (Fig 3).

There were no positive findings in the control group of 240 children 4–13 years of age (mean age 7.5 years, SD 2.5).

## Discussion

This study was the first to report the screening, diagnosis and estimation of prevalence of FXS among children attending special education in Sri Lanka. The screening was carried out in a systematic manner where the study population was initially screened for CGG repeat expansion, followed by further analysis for GZ, PM and FM in positive cases. 3’dTP-PCR and MCA was used for FXS screening because of its inherent advantage of being a single-step, closed-tube, homogeneous assay for rapid and large-scale screening for *FMR1* repeat expansion mutations in males and females, with high sensitivity from unmodified genomic DNA. The limitation of 3’dTP-PCR and MCA being a screening and not a diagnostic method was overcome by using CE, MS-PCR and Southern hybridization for characterization of mutations. Another special feature of the technique was the use of buccal cells as the primary source of DNA for initial screening, thus avoiding the need of blood samples.

This study gave a prevalence figure of 2.2% for CGG repeat expansions for children attending special education in Sri Lanka. The prevalence of FXS FM was 1.3%. A wide variation of prevalence figures for FXS is available from different parts of the world, among populations similar to our study. Figures available from Israel, Turkey, India and Saudi Arabia are 26.4%, 11.7% to 12.8%, 9.7% and 8.5% respectively [31–35]. In contrast, a lower figure of 0.25% is reported from the Atlanta, 0.5% from England and 0.8 to 2.4% from Japan [36–39]. We offer three possible reasons for this discrepancy in prevalence, which are relevant to our study as well. First is related to the techniques used for the analysis in different studies. For example, cytogenetic analysis in studies from Turkey and Greece has elicited high values of 11.7% and 6.5% respectively [32, 40]. On the contrary, Southern analysis for FXS among similar samples (mentally retarded individuals) in these two countries has elicited prevalence of 3% and 3.5% respectively [30, 41]. Second is the method of sample selection. Study populations have varied widely according to diagnostic categories of the subjects included. For example, an Indonesian study found a prevalence of 6 in 32 where there was a family history of FXS, but 1 in 144 in those with intellectual impairment alone [42]. A reported low prevalence of 1.1% from United States among children attending special education schools also included mentally retarded (12%), autism spectrum disorder (1%), learning disability (51%) and attention deficit hyperactivity disorder (35%) [43]. The relatively low prevalence is probably due to the high proportion of other disorders in the sample. The third reason is the variation in severity of intellectual impairment in the study samples. While some studies failed to detect FXS among individuals with borderline intelligence, samples with mild, moderate and severe disorders elicited prevalence of 2.1%, 9.65% and 3.4% respectively [30].

An additional finding of this study was the presence of methylated promoter C_P_G island in a PM sample having 140 repeats, contradicting the results reported by Devys *et al*., (1992) and Tassone *et al*., (2000) [44, 45]. These two studies reported that PM alleles have an unmethylated CpG island within the *FMR1* promoter.

A drawback is that other undiagnosed aberrations, which account for FXS, may have been missed due to the techniques used in this study. However, these aberrations (deletions and point mutations) account for less than 1% FXS frequency [46, 47]. Small sample size of 850 is also a drawback in comparison to some similar studies reported elsewhere [36]. Two studies from Asia have reported on small samples of less than 500 [48, 49]. However, the fact that no females with FXS were detected in our study may have been due to the small sample size.
